# Effectiveness of flipped classroom model in distance education: An experimental study in Turkish language course

**DOI:** 10.1371/journal.pone.0335721

**Published:** 2025-11-07

**Authors:** Seda Aktı Aslan, Yigit Emrah Turgut, Gökhan Ünlü, Jordan Allison

**Affiliations:** 1 Karakoçan Vocational High School, Fırat University, Elazığ, Türkiye; 2 Faculty of Education, Department of Computer Education & Instructional Technology, Recep Tayyip Erdoğan University, Rize, Türkiye; 3 Department of Computing Technologies, Munzur University, Cemisgezek Vocational High School, Tunceli, Türkiye; 4 School of Business, Computing and Social Sciences, University of Gloucestershire, Cheltenham, United Kingdom; Shandong University of Science and Technology, CHINA

## Abstract

This study investigates the impact of the Flipped Classroom Model (FCM) on university students’ learning achievement, self-directed learning skills (SLS), and course interaction within the context of live online classes (LOC). Employing a 14-week quasi-experimental design, the research involved an experimental group (n = 34) exposed to the FCM and a control group (n = 25) taught through traditional methods. Pre-tests indicated that the groups were not statistically different at baseline. Post-intervention analyses revealed no statistically significant differences between the groups in terms of immediate learning achievement or course interaction. However, results from a delayed post-test indicated that students in the experimental group demonstrated significantly higher retention of learning content compared to their counterparts. No significant improvements were observed in self-directed learning skills, suggesting that the FCM, as implemented, may not effectively foster such competencies across all learners. Furthermore, the model did not significantly enhance student interaction in the online classroom environment. These findings suggest that while the FCM may support long-term retention, its overall impact on achievement, interaction, and the development of SLS in LOC settings is limited and may be moderated by students’ existing self-regulatory capacities.

## Introduction

Information and communication technologies, whose role in educational environments is increasing significantly, offer numerous opportunities for enhancing learning and teaching processes. One such opportunity is the Flipped Classroom Model (FCM), which supports flexible learning using online learning materials. Through FCM, instructional content is delivered digitally outside the classroom, allowing in-person sessions to be dedicated to more student-centered learning activities [1]. FCM is increasingly implemented across diverse educational contexts. A notable variation of the traditional FCM is its adaptation to fully online settings, where even the in-class sessions are conducted in virtual environments through Live Online Classes (LOC) [2]. In this version, as with traditional FCM, students are expected to complete preparatory work using digital resources before synchronous sessions [3]. However, after completing the online lesson preparation process, students are not involved in face-to-face instruction as in traditional FCM, instead, students engage in collaborative and active learning activities within an online format [4]. This variation has been considered a practical approach to promoting active and collaborative learning [2].

As a reversal of the functioning of traditional teaching methods (TTM), FCM has been widely adopted in both K-12 and higher education settings [4,5]. Indeed, scholars have argued that it is important to adopt innovative approaches such as FCM that support students in actively constructing knowledge, as opposed to TTM, where students are passive recipients of the transmitted information [6–8]. The integration of FCM into LOC environments represents a meaningful pedagogical innovation. However, it has also prompted a growing body of research exploring its implications for various outcomes, particularly academic achievement, and its impact on learning processes [3,4,9,10]. Despite the increasing interest in FCM and its potential benefits, there remain concerns surrounding the implementation of FCM [11], especially in virtual formats, due to challenges such as increased student workload, variable student preparedness, and teacher difficulties in tracking engagement [12,13]. These challenges can negatively impact student outcomes [14], leading to mixed findings in the literature. While some studies report positive effects on academic performance [3,15,16], others suggest that it has no effect [17]. Therefore, this highlights the need for studies that consider FCM within LOC across different disciplines and student populations [18,19].

The FCM requires students to take responsibility and control for their learning [20]. The model’s success depends on students’ self-directed learning skills (SLS) [21]. In this context, students are expected to have SLS to prepare for the course content in the online environment and to complete individual or group activities in LOC [22]. However, not all students possess strong SLS, and those with limited capacities may struggle with preparation and effective participation [23,24]. Therefore, this variation in learner preparedness underscores the need to tailor the FCM to suit diverse learning styles and student needs [25]. Thus, FCMs inherent flexibility in allowing adaptations based on institutional goals and learner characteristics contributes to a lack of consensus in the literature regarding its effectiveness [26]. While some studies show that the model has a positive effect on students’ SLS [25,27] others report no effect [28,29]. Therefore, more research and evidence is required on the effects of the FCM on SLS [30].

Another key issue in the successful implementation of FCM is fostering meaningful student interaction. The model includes different various forms of student-centered interactions where students access content through video lectures in the online environment and group activities or discussions in LOC [31]. To optimize learning, it is necessary to support three distinct types of interactions in the model: content-student, student-student, and teacher-student. Each of which require different strategies than those used in traditional classrooms to support student interactions [32]. Particularly in online contexts where teacher presence may feel diminished, practitioners have developed models and systems to promote interaction and enhance student achievement [33–35]. Indeed, enriching the model in terms of interaction with such supports has yielded positive results for student learning [32,34]. Accordingly, the design of interactive environments, especially for LOC settings, warrants further investigation to enrich the theoretical and practical understanding of FCM.

This study aimed to determine the effect of the FCM in LOC on students’ (1) learning achievement, (2) self-directed learning skills, and (3) course interaction. To this end, this study addresses the following research questions:

1. What is the effect of using the FCM within LOC on learning achievement?
2. What is the effect of using the FCM within LOC on self-directed learning skills?
3. What is the effect of using the FCM within LOC on course interaction?

## Methods

### Participants and context

This study, which aims to reveal the effects of the FCM used in live online classes on students’ learning achievement, self-directed learning skills, and course interaction, was designed using a quasi-experimental design, one of the experimental research methods. The study was conducted with 59 undergraduate students at a public university, and the course subject was Turkish Language. The sample size was determined by the number of students available in the study setting and the resource constraints inherent to the implementation context, rather than through a priori power analysis. Details regarding the learning outcomes addressed during the experimental process are provided in Table 2. The reason why a quasi-experimental design is preferred is that groups cannot be formed randomly. In this study, intact class groups were used, meaning that one pre-existing class was assigned as the experimental group and the other as the control group. To ensure comparability between groups, pre-test scores were used, and based on the results, no significant differences were found between them. In cases where it is not possible to randomly form experimental and control groups, it is very important that the groups have similar characteristics [36]. Therefore, after selecting one of the groups as experimental and one as control, pre-tests were conducted to examine the groups’ scores on learning achievement and SLS. Detailed information on the scope and item analyses of the pre-test is presented in the “Learning Achievement Test” section. Before analyzing whether there was a significant difference between the groups’ pre-test scores, the data was examined to assess the assumption of normality. The Shapiro–Wilk test (p > .05) indicated that the data did not significantly deviate from normality, allowing it to be treated as approximately normal. Therefore, the independent groups t-test was performed. The results of the T-test are shown in Table 1.

**Table 1:** Pre-test score T-test results.

	Group	N	M	SD	*df*	*t*	*p*
**Learning Achievement**	Experimental Group	34	50.97	19.69	57	.930	.356
	Control Group	25	45.88	22.25			
**SLS**	Experimental Group	34	90.32	9.48	57	−.449	.655
	Control Group	25	91.52	10.90			

When Table 1 is examined, there is no significant difference between the groups’ pre-test scores for learning achievement (t(57)=.930, p > .05) and pre-test scores for SLS (t(57)=−.449, p > .05). Based on these results, the experimental and control groups did not differ significantly in their pre-test scores.

### Experimental procedure

**Fig 1:**
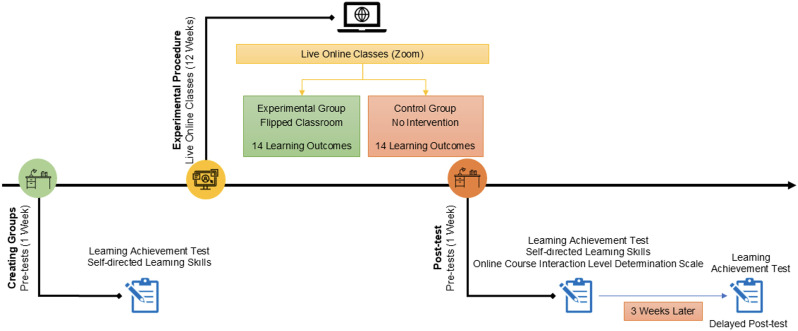
Experimental procedure.

This study followed a quasi-experimental design and the experimental process lasted 14 weeks, from September 23 to December 30, 2024. Participants were given general information and pre-tests in the first week of the experimental process. The information session included an overview of the study’s purpose, scope, and ethical considerations, such as voluntary participation and confidentiality, as well as the procedures to be followed during the research. Following this, written consent was obtained, and students signed consent forms to confirm their willingness to participate. During the next 12 weeks, the course’s learning outcomes were taught in both the experimental and control groups. A detailed list of the learning outcomes and topics covered in this process is given in Table 2. In the last week, the experimental process was completed with post-tests. In both groups, the classes were delivered as LOC via the Zoom application. While FCM was used in the experimental group, there was no intervention in the control group. The details of the classes in the experimental and control groups are given under the titles ‘Experimental process in the experimental group’ and ‘Experimental process in the control group.’ The experimental procedure is summarized in Fig 1.

**Table 2:** Distribution of test questions by learning outcomes and analysis.

Learning Outcomes	Question No	Item Difficulty	Item Distinctiveness
Find the predicate	36 37	0,36 0,40	0,65 0,55
Finding the subject	38 39	0,25 0,37	0,53 0,40
To find auxiliary verbs	41 42	0,42 0,41	0,66 0,41
Find the positive and negative clauses	31 35	0,43 0,63	0,58 0,74
Understanding the imperative, conditional, exclamation, and question clauses	11 13	0,81 0,51	0,83 0,44
Understanding the noun phrase	1 2	0,75 0,74	0,78 0,59
Understanding verb phrases	4 29	0,72 0,59	0,58 0,66
Understand a simple sentence	24 28	0,50 0,52	0,58 0,52
Understanding the compound sentence	15 16	0,51 0,62	0,47 0,37
Understanding an ordered sentence	19 32	0,30 0,67	0,64 0,59
Understanding the subordinate clause	17 23	0,29 0,33	0,47 0,78
Understanding the sentence	10 21	0,86 0,37	0,29 0,74
Understanding a simple sentence	7 26	0,68 0,55	0,68 0,67
To understand the sentence	18 22	0,64 0,63	0,50 0,74

### The design of the FCM activities in LOC

#### Experimental process in the experimental group.

In the experimental process, pre-tests were carried out in the first week, and then classes were taught in both groups for 12 weeks. The classes in the experimental group were based on FCM. Before starting the experimental process, the researchers prepared worksheets and videos of 6–7 minutes for each learning outcome. The videos were created by taking screen recordings of narrated PowerPoint presentations prepared by the researcher and included audio narration. In addition, worksheets were prepared for each outcome. Videos and worksheets were prepared for each of the 15 learning outcomes. In the videos, theoretical information about the learning outcome was presented shortly and concisely. The worksheets presented the subject’s main features and included the documents to be used in the collaborative activities to be carried out in class. The videos and worksheets were organized by considering the opinions of two experts in the field. The prepared videos and worksheets were evaluated by two academicians who are experts in the fields of instructional technologies and Turkish language education in terms of content appropriateness and pedagogical integrity. In line with the expert opinions, some necessary content and visual arrangements were made. The prepared documents were shared with the students weekly via a learning management system (Moodle). After the pre-tests in the first week of the experimental process, the students in the experimental group were informed about FCM. They were told how the class would be taught, where to access the course materials, and what to do before class. The information session was concluded by answering students’ questions about the subject. During the implementation process, care was taken to ensure that the extra-curricular activities were carried out efficiently by making reminders at the end of each class. In this process, the preparatory activities (watching videos and completing worksheets) that students were expected to perform before the lesson within the scope of FCM were followed up with reminders at the end of each week. In addition to in-class verbal reminders, weekly e-mail reminders were also sent to encourage preparation. Although these preparatory activities were not graded or formally assessed, the combination of monitoring via the Moodle LMS (tracking video views and worksheet downloads) and regular reminders indicated that nearly all students completed or accessed the preparatory materials each week. While the extra-curricular activities were carried out this way, cooperative learning activities were done in groups during the class. In these activities, attention was paid to forming heterogeneous groups. The groups were formed heterogeneously by taking into account the gender distribution and pre-test achievement scores of the students. Care was taken to include students from different achievement levels in each group. Group members were also encouraged to share responsibilities, take on leadership roles, and appoint spokespersons.

All classes were conducted as LOC using the Zoom application. The lessons were held twice a week, each session lasting 45 minutes. Breakout rooms were used for cooperative learning activities. Throughout the activities, the teacher regularly visited the breakout rooms, and care was taken to provide the necessary guidance to ensure that discussions were productive. Post-tests were administered in the last week of the experimental period. Three weeks after the post-tests, the process was completed with delayed post-tests.

#### Experimental process in the control group.

In the experimental process, as in the experimental group, 12 weeks of classes were taught after the pre-tests were administered in the first week. The following week, post-tests were conducted. The same measurement tool was used in the pretest, posttest and delayed tests applied to the control group. Delayed post-tests were administered three weeks after the post-tests. In the control group, as in the experimental group, all classes were delivered as LOC using the Zoom application. While FCM was used in the experimental group, there was no teacher intervention during the class in the control group. There was no external intervention in the teaching process in the control group. The teacher taught the lesson in accordance with her usual practices; no guidance or structuring was provided by the research team regarding the teaching method. It was observed that the teacher taught the class in the usual way, generally using lecture and question-and-answer techniques. In addition, sample questions related to the topics covered were solved at the end of each class. The experimental process was started and completed in the control group at the same time as in the experimental group.

### Data collection tools

#### Learning achievement test.

A learning achievement test was developed to measure students’ learning achievement in the control and experimental groups. This achievement test was originally developed by the researchers and was not adapted from any existing test. The test consisted of multiple-choice items. In developing the learning achievement test, care was taken to ensure it covered all 14 learning outcomes in the experimental process. First, a pool of 52 questions was created to cover all the learning outcomes. To calculate the item discrimination (r) and item difficulty (p) indices of the prepared questions, a pilot study was conducted with 250 university students. Item difficulty values between 0.30 and 0.70 were considered acceptable, with items below 0.30 classified as too difficult and items above 0.70 as too easy [37]. Item discrimination indices above 0.30 were considered acceptable to ensure that each item could adequately differentiate between high- and low-performing students [38]. As a result of the analyses, four questions with inappropriate item difficulty indices and ten questions with item discrimination indices below 0.30 were removed from the learning achievement test. Thus, the learning achievement test consisted of 28 questions, two for each of the 14 learning outcomes. The distribution of questions in the learning achievement test according to learning outcomes, item discrimination, and item difficulty index values is shown in Table 2.

The learning achievement test’s average discrimination was calculated as 0.55, its difficulty as 0.54, and the internal consistency coefficient (KR-20) as 0.75. The developed learning achievement test was used as a pre-test, post-test, and delayed post-test. In all three administrations (pre-test, post-test, delayed post-test), the same set of items was used. However, to minimize potential practice effects, the order of items was randomized in each administration and no feedback on correct answers was provided between tests.

#### Self-directed learning skills.

The SLS scale was developed by Aşkın [39]. The scale developed within a doctoral thesis’s scope aims to reveal university students’ SLS. The scale has a four-factor structure and consists of 21 items. The scale consists of four factors: ‘motivation, self-control, self-monitoring, and self-confidence.’ To provide a better understanding of what the scale measures in practice, one example item from each sub-dimension is as follows: ‘I enjoy learning’ (motivation), ‘I complete my learning tasks in a planned manner’ (self-control), ‘I evaluate my learning performance’ (self-monitoring), and ‘I am responsible for the decisions I make about learning’ (self-confidence). The reliability coefficients for each factor were 0.82, 0.80, 0.77, and 0.70, respectively. The reliability coefficient for the entire scale consisting of 21 items was calculated as 0.89 [39].

#### Online course interaction level.

The online course interaction level determination scale created by Erkal Karaman [40] was applied as a post-test to detect the interaction levels of the students in LOC. The five-point Likert-type scale consists of twenty-five items. Cronbach’s alpha reliability coefficient of the scale was determined to be 0.89. Additionally, the scale has been divided into four different sub-dimensions: “student, teacher, content, and presentation style” and the reliability coefficients of these sub-dimensions have been calculated as 0.92, 0.68, 0.66, and 0.46, respectively. To illustrate what each sub-dimension of the scale addresses, example items include: ‘Addressing students by their names’ (student), ‘Clear and understandable speech by the instructor’ (teacher), ‘Sharing documents and desktop content during the lesson’ (content), and ‘Active use of the whiteboard application’ (presentation style).

### Data analysis

To determine the appropriate analyses, the normality assumption was first assessed. All statistical analyses were performed on the raw data provided in S1 File. The Shapiro–Wilk test results indicated that a p-value greater than.05 suggested that the assumption of normality could be made and that the data were suitable for parametric tests [41]. As the data were assumed to be normally distributed, the independent samples t-test was used in the analyses, and the significance level was set at p < .05. The effect sizes (Cohen’s d) were interpreted based on the classification proposed by Cohen [42], where 0.20 is considered small, 0.50 medium, and 0.80 or above large.

Additionally, a post-hoc power analysis (two-tailed, α = .05) based on the observed effect sizes and group sizes (n₁ = 34, n₂ = 25) indicated an achieved power (1–β) of 0.55 for the delayed post-test (d = 0.56, 1–β = 0.55), 0.17 for the immediate post-test (d = 0.27, 1–β = 0.17), 0.09 for self-directed learning skills (d = 0.15, 1–β = 0.09), and 0.20 for course interaction (d = 0.30, 1–β = 0.20). These values reflect the exploratory nature of the study and the modest sensitivity to detect small effects.

## Findings

This part of the study is structured according to the research questions. The findings for each research question are presented under a separate title.

### Learning achievement (RQ1)

The first research question of the study is to examine the effect of the FCM used in LOC on student learning achievement. In this context, the post-test scores of learning achievement and delayed post-tests administered at the end of the experimental process were compared. Since the data were normally distributed, the independent group’s t-test was used to analyze the post-test scores obtained from the learning achievement test. The results of the analysis are presented in Table 3.

**Table 3:** T-test results of the groups’ learning achievement post-test scores.

Group	n	M	SD	*df*	*t*	*p*	*Cohen’s d*
**Experimental Group**	34	59.70	20.56	57	1.035	.305	.27
**Control Group**	25	54.04	21.05				

When Table 3 is examined, according to the independent groups t-test results of the groups’ learning achievement post-test scores, there is no significant difference between the groups (t(57)=1.035, p > .05). Although there was no significant difference between the learning achievement post-test scores of the groups, the mean scores of the experimental group (M = 59.70, SD = 20.56) were higher than the mean scores of the control group (M = 54.04, SD = 21.05). The effect size, as measured by Cohen’s d, was 0.27, indicating a small effect.

The delayed post-test was administered to students in both groups three weeks after the end of the experimental process to measure retention of acquired knowledge. The delayed post-test results were analyzed using an independent groups t-test, as the data showed a normal distribution. The results of the analysis are shown in Table 4.

**Table 4:** T-test results of the groups’ learning achievement delayed post-test scores.

Group	n	M	SD	*df*	*t*	*p*	*Cohen’s d*
**Experimental Group**	34	55.21	17.18	57	2.179	.033	.56
**Control Group**	25	44.30	21.24				

When Table 4 is examined, according to the independent groups t-test results of the learning achievement delayed post-test scores of the groups, there is a significant difference between the groups in favor of the experimental group (t(57)=2.179, p < .05).

When looking at the mean scores of the groups in the learning achievement delayed post-test, it is observed that the mean scores of the experimental group (M = 55.21, SD = 17.18) are higher than those of the control group (M = 44.30, SD = 21.24). The effect size for the difference between the groups, calculated using Cohen d, was 0.56, indicating a medium effect size.

### Self-directed learning (RQ2)

The study’s second research question is to examine the effect of the FCM in LOC on students’ self-directed learning skills. In this context, the SLS scale was administered to both groups at the end of the experimental process. As the data showed a normal distribution, the post-test scores obtained from the SLS scale were analyzed using an independent samples t-test. The results of the analysis are presented in Table 5.

**Table 5:** T-test Results of the groups’ self-directed learning skill post-test scores.

Group	n	M	SD	*df*	*t*	*P*	*Cohen’s d*
**Experimental Group**	34	91.57	7.72	57	−.574	.568	.15
**Control Group**	25	92.81	8.64				

According to Table 5, there is no significant difference between the groups according to the independent samples t-test results of the groups’ SLS post-test scores (t(57)=−0.574, p > .05). When the mean scores of the groups’ SLS post-test scores are examined, it is seen that the mean scores of the experimental group (M = 91.57, SD = 7.72) and the mean scores of the control group (M = 92.81, SD = 8.64) are close to each other. The effect size, as measured by Cohen’s d, was 0.15, indicating a small effect.

### Online course interaction (RQ3)

The third research question of the study is to examine the effect of the FCM used in LOC on course interaction. In this context, the course interaction scale was administered to both groups at the end of the experimental process. Since the data showed a normal distribution, the scores obtained from the scale were analyzed using an independent samples t-test. The results of the analysis are presented in Table 6.

**Table 6:** T-test results of the groups’ course interaction scores.

Group	n	M	SD	*df*	*t*	*P*	*Cohen’s d*
**Experimental Group**	34	56.96	8.44	57	−1.114	.270	.30
**Control Group**	25	59.23	6.67				

When Table 6 is examined, according to the results of the independent samples t-test on the groups’ course interaction scores, there is no significant difference between the groups (t(57)=−1.114, p > .05). When analyzing the mean scores of the groups’ course interactions, the mean scores of the experimental group (M = 59.23, SD = 6.67) are higher than those of the control group (M = 56.96, SD = 8.44). The effect size, as measured by Cohen’s d, was 0.30, indicating a small effect.

## Discussion

### The effect of the FCM used in LOC on learning achievement

Although the post-test achievement scores of the students in the experimental group were higher than those of the students in the control group, there was no significant difference between the two groups. This situation shows that the FCM used in LOC has a limited effect on improving student achievement. There are studies in the literature that support this finding [3,9]. The FCM in LOC supports student-centered learning and provides active learning experiences [2]. In addition, the model offers a wide range of applications by appealing to students with different learning styles [43]. In addition to these advantages, the model also has potential limitations, such as disengagement and distraction during online learning [44], lack of support [9], students feeling lonely [16], and increased student workload [45]. In the literature, it is noted that in the FCM used in LOC, students who have problems preparing and concentrating on the content before the lesson and have anxiety, particularly experience difficulties [16,46]. The fact that students experience such difficulties can impact their achievement [3].

The study found that the delayed post-test scores were statistically significantly higher in favor of the experimental group. In this sense, it was concluded that the FCM used in LOC had a positive impact on retention of acquired knowledge. As stated in the literature, it can be said that planning and designing the implementation process according to the needs of the learners is effective for this result [47,48]. In the study context, the presentation of practical activities that ensure active participation and support collaborative learning for the experimental group students may have made their retention of acquired knowledge. It is stated in the literature that applied and active learning has an impact on student achievement [49]. This impact can also be explained through several cognitive theories. According to the Testing Effect [50], repeated retrieval of information during active learning tasks strengthens memory traces and facilitates long-term recall. Similarly, the Generation Effect [51] suggests that producing information through problem-solving or collaborative tasks leads to deeper processing compared to passive reception. Within the framework of Cognitive Load Theory [52], pre-class engagement with concise videos and worksheets may have optimized intrinsic load and reduced extraneous load, allowing learners to allocate more cognitive resources to germane processing. Additionally, the use of both verbal explanations and visual materials aligns with Dual Coding Theory [53], which posits that integrating verbal and visual representations supports more robust memory formation. Together, these mechanisms may explain why retention improved more substantially in the FCM group compared to the control group.

### The effect of the FCM used in LOC on self-directed learning skills

The research found that the experimental and control groups’ post-test scores for SLS were close and not significantly different. Although some studies support this finding [54], some studies show that the model improves SLS [55]. Although the success of the FCM is based on students having SLS and using these skills in and out of the classroom [21,22], this is not enough to say that the model develops students’ SLS [29]. In fact, students’ low SLS and avoidance of taking responsibility are highlighted as important issues in using the FCM [56]. Especially LOC in FCM which student-teacher interaction is limited, students may not always be able to use effective learning strategies while working independently [17]. Furthermore, the literature suggests that students may have difficulties in organizing their learning in technology-supported environments [57] and that SLS should be supported for the effectiveness of online learning environments [58]. Indeed, students’ weak SLS may negatively affect their preparation for the course process and reflect on their performance [24]. At this point, the importance of providing students with appropriate guidance and immediate feedback is emphasized [56]. Furthermore, it is noted that self-orientation works in different ways for different students [23], and it is recommended that studies on this topic be expanded [30]. This finding is consistent with research indicating that the effective development of self-regulated learning skills requires showing students how to use these skills and incorporating structured support into the process [59,60]. Skills such as goal setting, effective time management, and self-monitoring may not automatically improve simply by participating in the FCM unless students are explicitly taught how to apply them step-by-step.

### The effect of the FCM used in LOC on course interaction

The study found no significant difference between students’ course interaction scores in the experimental and control groups. This result shows that FCM and LOC without any intervention affect students’ course interaction at a similar level. Interaction in online courses is an important factor affecting course quality, student success, and satisfaction [61,62]. In fact, it has been emphasized that online interaction effectively improves learning performance and outcomes in online environments [63] and encourages student participation [64]. At this point, it can be said that interaction in the FCM used in LOC is influenced by many factors and affects various factors [17]. However, the findings on the extent to which the FCM affects student interaction in LOC and which factors should be considered are limited in the literature [65]. Therefore, it can be said that more evidence is needed to promote student interaction and improve learning outcomes when implementing the FCM in online environments [66].

Indeed, there is no clear answer to how to create interactive structures in face-to-face classrooms and design an effective learning environment in the FCM used in LOC. From the perspective of the Community of Inquiry framework [67], structured opportunities for social presence and cognitive presence are crucial for fostering meaningful interaction. Although collaborative learning tasks were included in this study, the absence of structured interaction protocols—such as peer assessment, rotating discussion leadership, or guided reflection—may have limited opportunities for deep engagement [68,69]. This could explain why course interaction did not significantly improve despite the use of the FCM.

### Limitations

This study has several limitations. First, the sample size (n = 59) was relatively small, which may limit the statistical power and external validity of the findings. The sample size was determined based on the number of students available in the study setting and practical resource constraints, rather than through a priori power analysis. The quasi-experimental design without random assignments and the use of intact groups may increase the influence of certain uncontrolled variables. The scope of the study was limited to a Turkish Language course, and the intervention period was relatively short. Additionally, the internal consistency coefficient of the “presentation style” subscale of the interaction measure was relatively low (α = .46), which may have influenced the reliability of findings related to course interaction. Given these factors, the results should be interpreted with caution and considered exploratory in nature. Therefore, caution should be exercised when generalizing these results.

## Conclusion and implications

The findings of this study indicate that the Flipped Classroom Model (FCM), when implemented in online environments, can have a positive impact on retention of acquired knowledge. However, the model did not lead to significant improvements in self-directed learning skills or levels of course interaction. This suggests that the effectiveness of FCM may vary among learners, and students with lower self-directed learning skills may not benefit from the model to the expected extent.

These results emphasize the importance of incorporating supportive instructional designs that consider individual learner differences to enhance the effectiveness of FCM in synchronous online learning environments. In particular, integrating strategies aimed at fostering self-directed learning into the structure of the FCM may improve its overall impact.

By presenting experimental findings on the implementation of FCM in synchronous online course settings, this study contributes to the literature by shedding light on how various components of the model influence learning outcomes, especially within theoretical courses such as Turkish Language.

Future research is recommended to explore the application of FCM across different disciplines and with larger and more diverse student populations over extended periods. Additionally, evaluating FCM implementations enriched with self-regulatory learning strategies may provide further insights into enhancing the model’s effectiveness.

## Supporting information

S1 File Dataset. (CSV)
